# Treatment of Vogt-Koyanagi-Harada Disease

**DOI:** 10.7759/cureus.9125

**Published:** 2020-07-11

**Authors:** Musab K Alaql, Akinwale A Akinfe, Mohammed K AlNour

**Affiliations:** 1 Medicine, Jouf University, Sakaka, SAU; 2 Opthalmology, Prince Mutaib Bin Abdulaziz Hospital, Sakaka, SAU

**Keywords:** autoimmune, uveitis, systemic, granulomatous

## Abstract

Vogt-Koyanagi-Harada (VKH) disease is a T-cell-mediated autoimmune inflammatory disease characterized by granulomatous panuveitis with a variety of other systemic manifestations. A 29-year-old man referred with a two-week history of pain, redness, photophobia, and blurring of vision of the right eye. The patient reported a history of tinnitus and vertigo. Ocular examination revealed that the visual acuity was hand movement oculus dextrus (OD) and 1.0 oculus sinister (OS), slight periocular depigmentation in the right eye, iris bombe in the right eye, sunset glow sign similar to Dalen-Fuchs nodules of multifocal choroiditis in the right eye, reduced fovea reflex/subtle macular edema in the right eye, and normal anterior and posterior segment OS. The patient underwent a series of investigations and treatments, including corticosteroids, cyclosporine, antibiotics, and other local eye drugs. Surgical treatment included scheduling intravitreal ranibizumab for the right eye. Outcomes included improved general health conditions and improved visual condition (visual acuity improved to 0.8 OD). The combined therapy of immunosuppressive drugs with steroids was effective in improving visual impairment.

## Introduction

Vogt-Koyanagi-Harada (VKH) disease is an autoimmune inflammatory disorder that affects multiple systems with ocular, auditory, skin, and neurologic involvement [1]. It was described independently by Vogt, Koyanagi, and Harada as bilateral uveitis, exudative retinal detachments, integumentary disorders, and neurologic abnormalities [2]. Although uncommon, VKH represents 7% to 8% of patients with uveitis in Japan [3]. It is more common in Asian, Middle Eastern, Hispanic, and Native American populations [4]. An immunogenetic predisposition that is common in certain ethnic groups is more likely to be associated with VKH. The age of onset has ranged from 3 to 89 years, as studies reported, but the highest frequency was in people in their fourth decade of life [5,6].

Gender plays a role in the epidemiology of VKH. Women are twice as likely to be affected by VKH than men [7,8]. However, some other authors found that gender is not a risk factor for VKH [9,10]. We present a rare case of VKH disease illustrating that the combined therapy of immunosuppressive drugs with steroids was effective in improving the visual impairment.

## Case presentation

A 26-year-old man was referred with a two-week history of pain, redness, photophobia, and blurring of vision of the right eye. There was associated history of tinnitus and vertigo. There was no history suggestive of meningismus; however, there was malaise, fever, headache, nausea, abdominal pain, but no stiffness of the neck or back. There was slight periocular vitiligo, but no poliosis. There was no history of joint pain, and no history of oral or genital ulceration. There was no backache and no history of cough, night sweats, or chest pain. There was no prior history of ocular injury and no previous eye surgery. The patient was neither diabetic nor hypertensive. The review of systems was not contributory.

On general and systemic examination, there was no acute distress, and the man’s blood pressure was 131/68 mmHg. His pulse rate was 78 beats per minute. An ocular examination revealed that the visual acuity was hand movement oculus dextrus (OD) and 1.0 oculus sinister (OS), with a slight periocular depigmentation in the right eye, and ciliary injection in the right eye.

Applanation tonometry revealed 14 mmHg OD/16 mmHg OS. The examination also detected multiple moderate keratic precipitates in the right eye, moderate depth anterior chamber in the right eye, iris bombe in the right eye, posterior synechia with almost seclusio pupillae in the right eye, slight cortical opacities and clear vitreous in the right eye, sunset glow sign similar to Dalen-Fuchs nodules of multifocal choroiditis in the right eye, reduced fovea reflex/subtle macular edema in the right eye, and normal anterior and posterior segment OS.

The patient was admitted for proper evaluation and treatment with a multidisciplinary approach form of management.

Investigations included complete blood count, erythrocyte sedimentation rate, C-reactive protein, Mantoux test, venereal disease research laboratory, serial anterior segment photography, B-scan ultrasonography OD, brain computed tomography, orbit, and sinus with contrast, lumbar puncture for cerebrospinal fluid (CSF) pleocytosis, computerized visual field, optical coherence tomography, fundus fluorescein angiography/indocyanine green, and an electroretinogram.

Treatment aimed to minimize the patient’s symptoms and save his eyesight through suppression of the ciliary spasm resulting in photophobia OD, suppression of the anterior segment inflammation OD, adhesiolysis OD, and treatment of macular edema and features suggestive of choroiditis. Medical treatment included intravenous (IV) methylprednisolone 1 g in 250 ml saline given over 30 minutes per day for three days and then tablet prednisolone 15 mg three times per day for three days, 10 mg three times per day for three days, and 5 mg three times per day for three days; IV ceftriaxone 1 g every 12 hours for three days, IV metronidazole 500 mg every eight hours for three days, IV omeprazole 40 mg daily for three days, Gutt prednisolone forte every two hours OD for seven days, Gutt Vigamox every four hours OD for seven days, Gutt Ocugesic every six hours OD for seven days, Gutt cyclosporines 0.05% (Restasis) every eight hours OD for seven days, and Gutt atropine every 12 hours OD for seven days.

**Figure 1:**
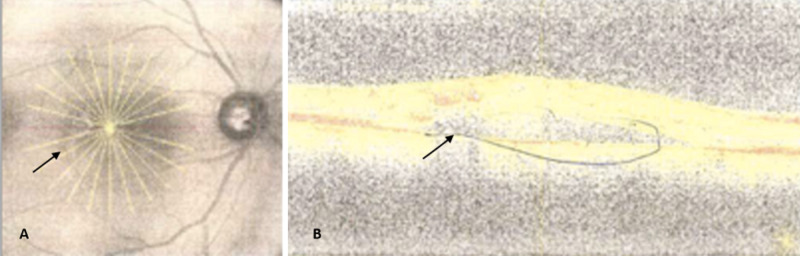
(A) Right eye optical coherence tomography with obvious darkening of the fovea aspect of the macular and disc pallor. (B) Obvious early onset cystoid macula edema oculus dextrus.

Surgical treatment included an intravitreal ranibizumab injection for the right eye after obtaining informed consent for an intravitreal anti-vascular endothelial growth factor injection for the right eye (Figure 1).

Outcomes included improved general health and improved visual conditions (visual acuity improved to 0.8 OD).

## Discussion

VKH disease is characterized by bilateral uveitis as the most common initial manifestation with red eyes, blurred vision, and pain. This is usually accompanied by systemic symptoms. Auditory symptoms include vertigo, tinnitus, and deficient hearing. Neurologic symptoms include meningeal irritation and involve headache, stiffness of the neck and back, meningitis, CSF pleocytosis, cranial nerve palsies, hemiparesis, transverse myelitis, and ciliary ganglionitis. Cutaneous manifestations include poliosis, vitiligo, and alopecia. The vitiligo often is found in the sacral region [2].

The broad spectrum of symptoms found in VKH suggests that there is a central mechanism involved in the multisystemic manifestations. Currently, VKH is believed to be a T-cell-mediated autoimmune disease [11]. An antigenic component present in dermal, uveal, and meningeal melanocytes seems to be directing an autoimmune reaction. More evidence suggesting that VKH is an autoimmune disease is its association with other autoimmune disorders. These include Hashimoto thyroiditis, autoimmune polyglandular syndrome, immunoglobulin A nephropathy, and Guillain-Barré syndrome [12-15].

Clinically, VKH can be divided into four stages: prodromal, acute uveitic, convalescent, and chronic recurrent [16]. In the prodromal phase, symptoms may mimic a viral infection presented with flu-like symptoms that lasts for a few days [17]. The acute uveitic stage occurs within three to five days of the first stage as patients may experience blurred vision in both eyes due to diffuse choroiditis [17]. The convalescent stage usually follows a few months later. At this stage, depigmentation of the integument and choroid occurs [18]. The chronic stage may develop by interrupting the convalescent stage in 17% to 73% of patients [16,17]. Ocular complications such as cataract, choroidal neovascularization, glaucoma, and retinal fibrosis can be observed in this stage [8].

This patient’s visual prognosis is generally good with prompt diagnosis and aggressive immunomodulatory treatment. Response of the inner ear to corticosteroids take one to four months and can give complete improvement of hearing, but corticosteroids cannot guard against effects on the eye, and it may lead to cataract, glaucoma, and optic atrophy. Skin changes typically persist despite therapy.

## Conclusions

In this rare case of VKH disease, the combined therapy of immunosuppressive drugs with steroids was effective in improving our patient’s visual impairment. A holistic , multidisciplinary approach to health care is warranted to optimize patient outcomes.
